# Survey of farm, parlour and milking management, parlour technologies, SCC control strategies and farmer demographics on Irish dairy farms

**DOI:** 10.1186/s13620-024-00267-y

**Published:** 2024-05-06

**Authors:** Alice Uí Chearbhaill, Pablo Silva Boloña, Eoin G. Ryan, Catherine I. McAloon, Alison Burrell, Conor G. McAloon, John Upton

**Affiliations:** 1 Teagasc, Animal & Grassland Research and Innovation Centre, Moorepark, Fermoy, Co. Cork P61 C997 Ireland; 2 School of Veterinary Medicine, University College Dublin, Belfield, D04 W6F6 Dublin 4 Ireland; 3 Animal Health Ireland, 2–5 The Archways, Carrick On Shannon, N41 WN27 Co. Leitrim Ireland

**Keywords:** Parlour technologies, Milking management, Farm management, SCC control

## Abstract

**Background:**

This cross-sectional study describes a survey designed to fill knowledge gaps regarding farm management practices, parlour management practices and implemented technologies, milking management practices, somatic cell count (SCC) control strategies, farmer demographics and attitudes around SCC management on a sample of Irish dairy farms.

**Results:**

We categorized 376 complete responses by herd size quartile and calving pattern. The average respondent herd was 131 cows with most (82.2%) operating a seasonal calving system. The median monthly bulk tank somatic cell count for seasonal calving systems was 137,000 cells/ml (range 20,000 – 1,269,000 cells/ml), 170,000 cells/ml for split-calving systems (range 46,000 – 644,000 cells/ml) and 186,000 cells/ml for ‘other’ herds (range 20,000 – 664,000 cells/ml). The most common parlour types were swing-over herringbones (59.1%) and herringbones with recording jars (22.2%). The average number of units across herringbone parlours was 15, 49 in rotary parlours and two boxes on automatic milking system (AMS) farms. The most common parlour technologies were in-parlour feeding systems (84.5%), automatic washers on the bulk tank (72.8%), automatic cluster removers (57.9%), and entrance or exit gates controlled from the parlour pit (52.2%). Veterinary professionals, farming colleagues and processor milk quality advisors were the most commonly utilised sources of advice for SCC management (by 76.9%, 50.0% and 39.2% of respondents respectively).

**Conclusions:**

In this study, we successfully utilised a national survey to quantify farm management practices, parlour management practices and technology adoption levels, milking management practices, SCC control strategies and farmer demographics on 376 dairy farms in the Republic of Ireland. Rotary and AMS parlours had the most parlour technologies of any parlour type. Technology add-ons were generally less prevalent on farms with smaller herds. Despite finding areas for improvement with regard to frequency of liner changes, glove-wearing practices and engagement with bacteriology of milk samples, we also found evidence of high levels of documentation of mastitis treatments and high use of post-milking teat disinfection. We discovered that Irish dairy farmers are relatively content in their careers but face pressures regarding changes to the legislation around prudent antimicrobial use in their herds.

**Supplementary Information:**

The online version contains supplementary material available at 10.1186/s13620-024-00267-y.

## Background

Dairy farming contributes hugely to the Irish economy, providing €16 billion of economic value and around 85,000 jobs [1]. In order for the Irish dairy sector to remain competitive in the highly risk-sensitive global food market, it is imperative that animal health and milk quality are prioritised and optimised [2]. Mastitis, defined as inflammation of the mammary gland, is one of the greatest economic costs to dairy farmers [3, 4]. A bulk tank somatic cell count (BTSCC) below 400,000 cells/ml is currently the minimum requirement when supplying milk for human consumption according to European Law [5]. Irish legislation defines an ‘SCC breach’ as a geometric mean SCC value exceeding 400,000 cells/ml, based on all sample results over the previous three-month period, with at least one sample per month [6]. A further three-month recovery period is provided for corrective action and, if the bulk tank geometric mean SCC still exceeds 400,000 cells/ml, deliveries of milk from that holding must be suspended [6]. A threshold of 200,000 cells/mL is used at the herd level where BTSCC > 200,000 cells/ml is often suggestive of a subclinical mastitis problem in the herd [7]. However, it can also indicate contamination of the bulk tank with high SCC milk from lack of identification of clinical mastitis cases [8]. Recent estimates are that 65% of Irish dairy farms have an unadjusted geometric mean BTSCC of < 200,000 cells/ml [9]. Milk processors increasingly apply incentives and penalties across a wide range of different milk quality parameters, so it is of utmost importance to producers to maintain low levels of BTSCC [10].

In 2022, the number of dairy cows in the Republic of Ireland amounted to 1.51 million [11], collectively producing 8.8 billion litres of milk [12]; an increase of 7.9% and 20.5% respectively on 2017 figures [13]. In general, as dairy farms grow larger, staff time spent per cow decreases and the throughput of cows at milking increases. Farmers who monitored and participated in milking themselves were found to be an important factor associated with reduced BTSCC [7]. With increasing herd size, there is a pressing need for improved parlour efficiency on Irish dairy farms [14, 15]. However, it is imperative that any future changes in herd size and its impact on parlour efficiency do not contribute to compromised udder health or general husbandry of individual animals. The Irish dairy production system is one of mostly seasonal, pasture-based dairy production to suit the temperate climate [16]. In an Irish study by O’Donovan et al. [17], milking (including herding the cows to and from the parlour, milking and washing the parlour after the milking process) accounted for 34% of average annual dairy labour input for an average herd size of 77 cows. As labour accounts for one of the highest costs of pasture-based systems [18], technology can be adopted to automate some of the more mundane tasks of dairying. In fact, a reduction of labour is likely the key motivator for farmers to adopt automation technologies [19].

Work practices and technologies that are known to offer the largest labour savings for milking include having one person in the milking pit during mid lactation (i.e. one person conducting and observing milking), with added benefit if entrance or exit gates can be controlled from the pit, and automatic cluster removers (ACRs) [20]. Automation of post-milking teat disinfection may award farmers more time for observing and ensuring proper attachment of clusters on un-milked cows; an intervention which would be of benefit to the overall udder health of the herd if automated to perform in an optimal manner [21, 22]. Other technologies most commonly adopted by Irish dairy farmers include automatic parlour feeders, milk meters and automatic washers of the milking machine and bulk tank [23].

There is a lack of data in the Republic of Ireland on which are the most common milking practices. Internationally, it has been established that good milking management practices are associated with reduced BTSCC [7, 24–26]. Both pre- (washing and/or drying, stimulation and disinfection of teats) and post-milking management practices (disinfection of teats following teat cup removal and rinsing or flushing of clusters) influence the likelihood of contamination of teats with mastitis-inducing pathogens before, during and after the milking process [27]. Therefore, with increased pressure on farmers to reduce their reliance on antibiotic use [28], ensuring hygienic milking practices [26, 29, 30] and appropriate adoption of parlour technologies is imperative. In addition to milking management practices, studies have shown that self-reported farmer attitudes and behaviour can account for as much as 48% of the variation in BTSCC between herds [31].

The objective of this cross-sectional study was to document the farm management, parlour management including parlour technologies, milking management practices, SCC control strategies and farmer demographics on a sample of commercial Irish dairy farms. The resulting survey database, consisting of milk quality data, farm technology and farm management data, is described in the current paper. The database will be used to assess the impacts of various technologies and management practices on BTSCC in subsequent work.

## Materials and methods

### Farm technology and management survey

#### Survey design

A survey was developed and hosted on the SurveyMonkey online platform (Momentive Global Inc., CA USA). The survey was developed in collaboration with a subset of the Animal Health Ireland (AHI) CellCheck Technical Working Group, ensuring systematic development of each section in accordance with the study objectives. Relevant experts and stakeholders were consulted and feedback was acquired at multiple stages throughout the development process. The survey was circulated to experts in the fields of academia, veterinary practice, and behavioural science to ensure that it was fit for purpose in answering the study objectives. The survey was also scrutinised using cognitive interviews with six commercial dairy farmers who had never been exposed to the survey previously and provided feedback and insight into how it would be interpreted by the target audience. Inclusion of a question about whether farms were managed as a partnership was included in the survey as a result of these cognitive interviews. The survey was created in a format compatible with both desktop and mobile devices, and followed Dillman’s tailored design survey protocols [32].

The survey consisted of 66 questions across 13 pages with a mixture of multiple choice, check-box, dropdown menu, rating scale and ‘textbox’ questions. It was divided into five sections pertaining to (i) general contact information, (ii) farm-specific management, (iii) parlour-specific management, including parlour technologies, (iv) cow-specific management, including milking management and SCC control strategies, and (v) farmer-specific questions. Mandatory consent was obtained for sharing herd milk recording, bulk milk and stock data via the Irish Cattle Breeding Federation database (ICBF, https://www.icbf.com/).

The farm management section included information on parlour type, parlour manufacturer, mastitis treatment records, numbers of milking cows in 2021 and 2022, frequency of milking and the number of cows culled in 2021 specifically for high SCC. The parlour management and parlour technology information section included questions regarding the normal morning and evening milking times and durations, the number of people milking during peak lactation and the relation of these people to the farmer, the age and characteristics of the milking system, information on technological add-ons, parlour servicing, frequency of liner changes and cluster disinfection practices. The SCC and milking management section included information on fore-milking, California mastitis testing (CMT), pre- and post-milking management, teat disinfection products, glove-wearing practices and antibiotic and teat sealant application during the 2021 dry-off season. The farmer-specific section included questions about their gender, age, level of education, years spent in the dairy industry, and information regarding their personal feelings towards udder health problems, such as SCC on their farms, who they obtain SCC advice from, their attitudes towards the changing legislation on antibiotic usage at dry-off and their overall satisfaction with the profession of dairying.

A full list of questions and number of responses to each question can be found in Supplementary Materials 1.

### Survey circulation

Communications were made via phone and email with members of major Irish milk processors (Arrabawn, Aurivo, Bandon, Barryroe, Clonakilty, Centenary Thurles, Dairygold, Drinagh, Tirlán, Lakeland, Limerick Liquid Milk Producers, Lisavaird, Mullinahone, Kerry, Tipperary). The survey link was circulated to all of their suppliers by text message. The survey was circulated in July 2022 and farmers were given two months to respond. The circulation population considered for the survey were approximately 15,300 specialist dairy farms across 26 counties in the Republic of Ireland [33].

In total, 666 dairy farmers responded to the survey. Of this, 432 respondents fully completed the survey; the remainder submitted surveys which were partially completed. Complete surveys accounted for 64.9% of the total survey responses. The average time spent completing the survey was 22 min and 46 s.

### Farm production and BTSCC data

Monthly bulk tank data from January 2021 to August 2022 (processor name, milk supplied in litres (L), fat (%), protein (%), bulk tank somatic cell count (BTSCC, × 1000 cells/ml), total milk solids (kg), and total number of dairy cows) were requested from the ICBF database for the 432 farmers who completed the survey. These data were acquired from respondents’ respective milk processors. For the purpose of this paper, these data will be referred to as ‘processor data’. Mandatory consent was acquired for this from all respondents at the beginning of the survey. Without granting consent, farmers were unable to access the survey.

### Data pre-processing

Data collected from the online survey were exported to spreadsheets for analysis. Responses from the survey were individually reviewed, and answers which were incomplete or implausible were identified and removed (see data removal). All addresses were inspected and awarded a general county-based data label. Any farms which did not offer adequate address details or postal codes were input into ICBF using their herd number and their county of occupancy was extracted. Specific address information was removed from the dataset prior to analysis.

### Data removal

Cleaning of processor data was conducted to identify errors. Three farms were identified as supplying milk to more than one processor and therefore had duplicate milk data. Data for both processors were combined into one record; the data for most columns were identical and averages of the two values were taken where this was not the case. These processor data were then merged with the survey data.

Of the 432 completed responses, 34 farms were removed due to inadequate herd number or contact information in their survey response preventing the extraction of their information on ICBF, 14 were removed as there was no processor data provided to correspond to their survey answers, and one was removed due to inappropriately answered survey questions. Two farms responded to the survey twice. The most recent response for each of these herd numbers was taken as the final response. A total of 376 herds in the dataset supplied milk for 2021 and 381 supplied milk in 2022. Only herds present in the dataset across both years were included in the final analysis, hence, our final dataset contained 376 herds (7,090 monthly observations). A total of 13.0% of respondent herds were removed from the survey dataset during this processing step.

We checked the monthly values for BTSCC that would fall outside the parameters set by O’Connell et al. [34]. These included herds that supplied milk for less than six months of the year (though we corrected this to less than four months for 2022 given that we only had data for eight months), monthly BTSCC values of < 20,000 cells/ml and monthly milk volumes of < 227.5 L (corresponding to the minimum milk volume collected by milk processors in Ireland). No records were removed for herds milking less than six (less than four for 2022) months of the year nor for having an SCC < 20,000 cells/ml, though two farms had one month each where SCC was 20,000 cells/ml exactly. Removing records below the minimum monthly milk collection volume reduced the number of monthly observations by 5.8%.

### Data processing

Data were processed using SAS OnDemand for Academics (https://welcome.oda.sas.com/). Herds were identified as seasonal calving, split calving or ‘other’ as per O’Connell et al. [34]. Seasonal calving herds were defined as herds which calved all cows between February and April and peak milk production occurred in May or June and exceeded the herd’s minimum monthly milk production in the herd year by > 700%. Split calving herds supplied milk throughout December and January and had peak milk production that exceeded herd minimum milk production for any month by < 300%. Any herds that did not meet either of these requirements were classified as ‘Other’.

Using the PROC Univariate procedure (SAS OnDemand), herd size quartiles (Q1, Q2, Q3, Q4), from the average total dairy cow numbers of 20 months data (i.e. average number of lactating animals of parity one or greater), were determined from the monthly processor values.

It is important to note that there was no obligation for respondents to answer every question, resulting in varying levels of response rates per question. All percentage response figures presented in the results were calculated on a question-by-question basis. A full list of questions and relative responses can be found in Supplementary Materials 1. Some questions had an ‘other’ or manual text box input option and these are generally not specifically mentioned in the results section in the interests of highlighting the main survey results.

## Results

### Respondent overview

**Fig. 1:**
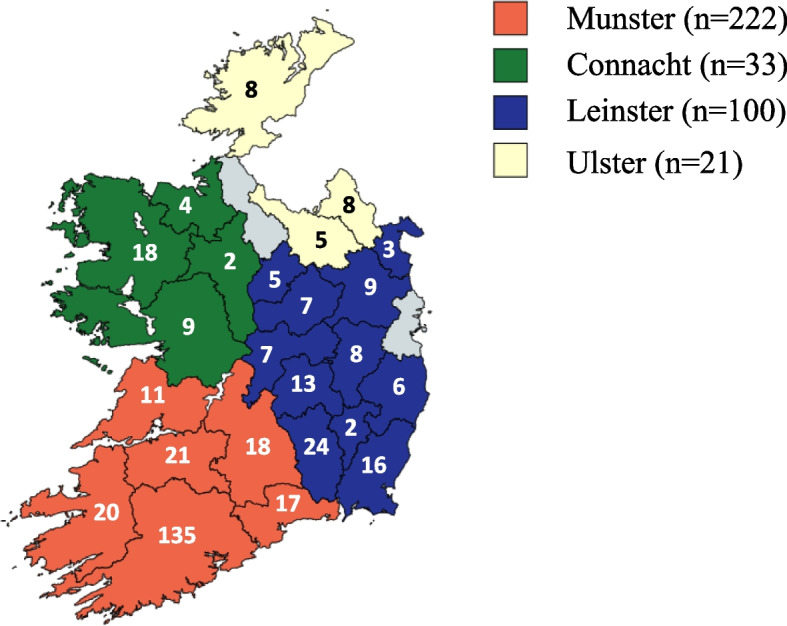
Distribution of survey respondents by county

The geographical distribution of survey respondents across 24 out of 26 counties in the Republic of Ireland can be observed in Fig. 1.

The average herd size of respondent farms was 131 cows (Table 1). Quartiles by herd size were as follows; Q1 herds had an average of 55 cows (range 9–73), Q2 herds had an average of 88 cows (range 74–105), Q3 herds had an average of 127 cows (range 105–159) and Q4 herds had an average of 253 cows (range 159–847), see Table 2.

**Table 1:** Monthly average farm bulk milk production data for 12 months of 2021, 8 months of 2022 and combined 20 months (from processor dataset)

	**2021**		**2022**		**Combined**	
**Variable Label**	**Mean / Median**^a^	**Std Dev / IQR**^b^	**Mean / Median**^a^	**Std Dev / IQR**^b^	**Mean / Median**^a^	**Std Dev / IQR**^b^
SCC (× 1000 cells/ml)	150	103	137	108	145	105
Milk volume (L)	65,082	56,031	75,159	60,745	69,087	58,155
Fat %	4.5	0.5	4.2	0.4	4.4	0.5
Protein %	3.7	0.3	3.5	0.2	3.6	0.3
Total Milk Solids (kg)	5,332	4,561	5,983	4,919	5,591	4,717
Total Dairy Cows	129	92	134	96	131	93

^a^Median values for SCC

^b^Interquartile range values for SCC

**Table 2:** Monthly average farm bulk milk production data by herd size quartiles across 12 months of 2021 and 8 months of 2022 combined (from processor dataset)

Average Annual Herd Number Quartiles	Q1 < 73 cows				Q2 cows				Q3 106–159 cows				Q4 > 159 cows			
Variable Label (Monthly Average Values)	Mean / Median^a^	Std Dev / IQR^b^	Min	Max	Mean / Median^a^	Std Dev / IQR^b^	Min	Max	Mean / Median^a^	Std Dev / IQR^b^	Min	Max	Mean / Median^a^	Std Dev / IQR^b^	Min	Max
SCC (× 1000 cells/ml)	134	119	20	1,269	139	110	20	1,072	149	102	29	1,006	152	91	41	770
Milk volume (L)	28,091	15,939	313	78,751	47,419	21,919	230	118,021	68,170	32,260	372	149,420	131,490	75,141	334	554,816
Fat %	4.3	0.4	3.4	6.9	4.3	0.4	3.4	6.2	4.4	0.5	3.4	6.2	4.5	0.5	3.4	6.5
Protein %	3.5	0.3	2.9	4.9	3.6	0.3	2.9	4.7	3.6	0.3	3.0	4.8	3.7	0.3	3.1	4.7
Milk Solids (kg)	2,231	1,224	26	5,854	3,774	1,671	20	9,442	5,486	2,457	31	11,693	10,774	6,066	29	46,617
Total Dairy Cows ^c^	55	16	9	73	88	9	74	105	127	15	105	159	253	105	159	847

*n* = 376; 1,743 herd observations for Q1, 1,779 herd observations each for Q2 and Q3, 1,789 herd observations for Q4

^a^Median values for SCC

^b^Interquartile range values for SCC

^c^Annual average values; 187 herd observations for Q1, 188 herd observations each for Q2 and Q3, 189 herd observations for Q4

### Production and BTSCC data

**Fig. 2:**
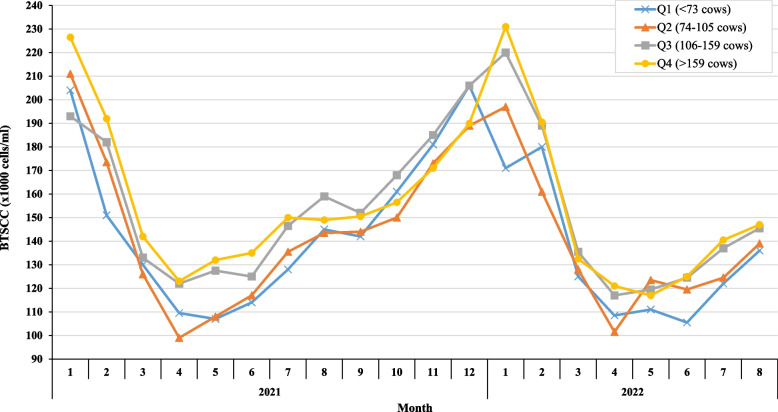
Median monthly BTSCC by herd size quartiles across 12 months of 2021 and 8 months of 2022 (from processor dataset) (*n* = 376)

Table 1 shows the average monthly milk production and BTSCC for 2021, 2022 and for both years (20 months) combined (*n* = 376). For the 20 months combined, monthly average herd milk production was 5,591 kg of milk solids and average monthly BTSCC was 145,000 cells/ml (Table 1). Milk production data by herd size quartiles can be observed for both years combined in Table 2. Figure 2 shows the temporal trends in monthly BTSCC by herd size quartile showing nadir BTSCC from April to June each year and a rise in BTSCC from September–October onwards.

### Seasonality

**Fig. 3:**
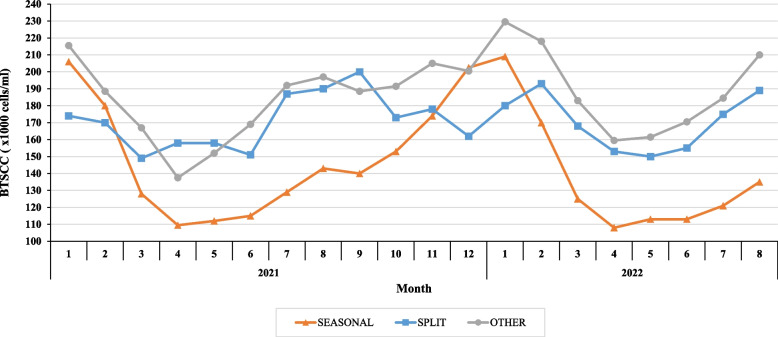
Median monthly BTSCC by calving patterns across 12 months of 2021 and 8 months of 2022 (from processor dataset) (*n* = 376). ‘SEASONAL’ refers to herds which calved all cows between February and April and those in which peak milk production occurred in May or June and exceeded the herd’s minimum monthly milk production in the herd year by > 700% (*n* = 309). ‘SPLIT’ refers to herds which supplied milk throughout December and January and had peak milk production that exceeded herd minimum milk production for any month by < 300% (*n* = 29). ‘OTHER’ calving patterns are milk producers which do not fit into either of the two former categories due to the limitations set in characterising them (*n* = 38)

**Fig. 4:**
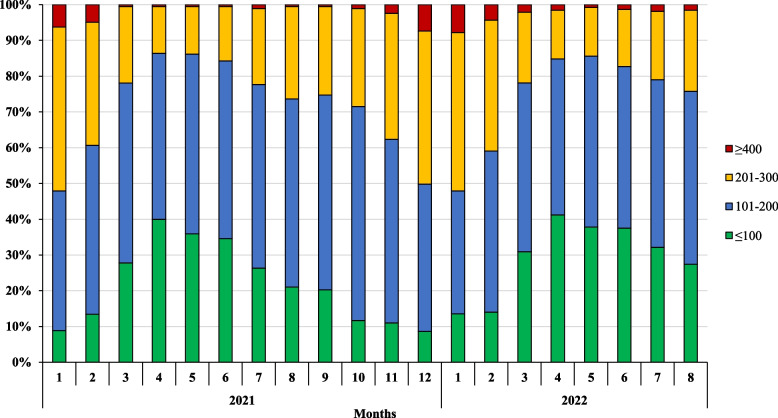
Percentage of respondent herds with monthly BTSCC equal to or below 100,000 cells/ml, between 101–200,000 cells/ml, between 201–399 cells/ml and equal to or above 400,000 cells/ml across 12 months of 2021 and 8 months of 2022 (from processor dataset) (*n* = 376)

We identified 309 herds as seasonal calving, 29 herds as split-calving and 38 herds as ‘other’. Figure 3 shows the temporal trends in average monthly BTSCC by calving pattern. The median monthly bulk tank somatic cell count for seasonal calving systems was 137,000 cells/ml (range 20,000 – 1,269,000 cells/ml), 170,000 cells/ml for split-calving systems (range 46,000 – 644,000 cells/ml) and 186,000 cells/ml for ‘other’ herds (range 20,000 – 664,000 cells/ml). Median monthly BTSCC varied across all seasonal-calving herds from a minimum of 108,000 cells/ml in late spring/early summer to a maximum of 209,000 cells/ml in autumn/winter. In contrast, median monthly BTSCC fluctuated between 149,000 cells/ml and 200,000 cells/ml across all split-calving herds and between 137,500 cells/ml and 229,500 cells/ml across all ‘other’ herds. Figure 4 shows the percentage of herds with a monthly BTSCC equal to or below 100,000 cells/ml, between 101–200,000 cells/ml, between 201–399,000 cells/ml and equal to or above 400,000 cells/ml across 12 months of 2021 and 8 months of 2022. The percentage of herds with a BTSCC of ≥ 400,000 cells/ml was greatest in the months of January (6.3%), February (4.9%) and December (7.4%) for 2021 and January (7.8%) and February (4.3%) of 2022. A monthly BTSCC of < 100,000 cells/ml was most commonly achieved in the months of April (40%; 41.2%), May (35.9%; 37.8%) and June (34.6%; 37.5%) for 2021 and 2022, respectively.

### Farm technology and management survey

#### Farm management

Herds managed as part of a partnership accounted for 22.3% of herds and 3.7% of respondents milked in more than one parlour over the course of lactation. Most respondents milked twice per day (95.2%), 1.3% of respondents milked once per day, and 0.8% responded with ‘other’. The ‘other’ answers pertained to different milking practices at different times of the year, for example “13 milkings [from] July to Dec” and “do 10 in 7 [days] for the last three months of 2021”. Respondents that milked with an AMS and therefore had no set milking schedule accounted for 2.7% of responses.

Morning milking most commonly started between 7 and 8am (61.0% of respondents) and most commonly lasted 90 min (25.2%). Evening milking start time most commonly occurred between 4 and 5 pm (55.0%) and lasted 90 min (30.0%). The most common interval between morning and evening milkings was 9 to 10 h (60.1%). Most commonly, there was only one person milking in the parlour for morning (68.8%) and evening (66.5%) milkings. During milking, 90.7% of the survey respondents were present at the milking themselves, 57.1% enlisted a family member and 33.6% hired an employee. Most of the milking parlours were less than 10 years of age (46.8%), with 20.1% of these being less than 5 years of age. Upgrades were applied to parlours in the past five years on 36.8% of respondent farms.

Farmers were asked for their most likely influencing factors for culling on a 7-point scale (‘1’ being most likely reason to cull and ‘7’ being least likely reason to cull). Fertility problems were given a score of ‘1’ or ‘2’ by 50.2% of respondent farms, as was persistent high SCC (45.0%) and recurrent clinical mastitis (40.7%). Lameness and poor milk production were awarded mid-level scores of ‘3–5’ by 60.8% and 57.6% of respondent farms respectively. A score of ‘6’ or ‘7’ was awarded to individual cow behaviour by 56.0% and age by 53.4% of respondent farms.

#### Parlour management and technologies

The most prevalent parlour types were swing-over herringbones (59.1%) and herringbones with milk recording jars (22.2%). This was followed by parallel parlours (6.7%), double-up herringbones (4.8%), rotaries (2.7%) and AMS (2.7%). Less frequent parlour types submitted as an ‘other’ option included one abreast parlour, one bucket plant, two herringbones with no recording jars, one parallel parlour with recording jars, one non-descript herringbone and a rapid exit parlour. The average number of units across the various types of herringbone parlours was 15 (range 2–40), 49 in rotary parlours (range 40–70) and two boxes on AMS farms (range 1–3). Most parlours had a mid-level milk line (90.1%). The most common pulsation system was an alternating system (53.9%) followed by simultaneous (41.3%) and ‘one pulsator per teat’ systems (3.8%).

One parlour service per year was carried out on 72.1% of respondent farms, 15.2% serviced twice per year, 8.0% serviced less than once per year and 4.8% serviced more than twice per year. Liner changes occurred most commonly once (34.4%) or twice (44.8%) per year. Other variations of liner change frequency included every 2,000 milkings (6.7%), every 2,500 milkings (6.1%), three times per year (4.5%) or every two years (1.3%).

Farmers had various means of ensuring good cow positioning in the parlour. A full list of cow positioning additions by parlour type can be found in Supplementary Table 6 and a list of total positioning additions by parlour type in Supplementary Table 7. Individual mangers were present in 59.6% of parlours. Straight rump rails were present in 39.3% of parlours whilst 14.8% had zig-zag rump rails. Manual bailing systems were present in 13.7% of respondent parlours whilst 5.5% had a sequential bailing system. Straight breast rails and adjustable breast rails were present in 7.1% and 8.5%, respectively, of respondent parlours.

**Fig. 5:**
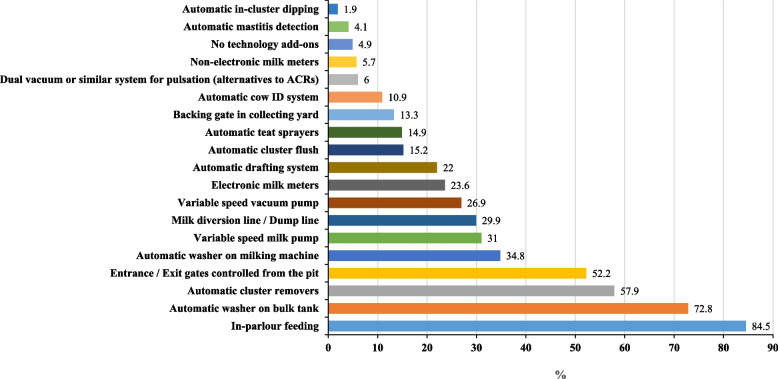
Total % of parlour technological add-ons across all parlour types (*n* = 368)

Farmers also had varying degrees of technological additions in their parlours. A full list can be observed in Fig. 5. A full list of parlour technology additions by herd size quartiles can be found in Table 3 and Supplementary Table 4. A full list of parlour technology additions by parlour type can be observed in Supplementary Table 8 and a list of total technology additions by parlour type in Supplementary Table 5. No technological add-ons were reported in 4.9% of respondent farms. For those that did have them, the most common add-ons were in-parlour feeding systems (84.5%), automatic washers on the bulk tank (72.8%), ACRs (57.9%), entrance or exit gates controlled from the pit of the parlour (52.2%), and automatic washers on the milking machine (34.8%). Other add-ons included variable speed milk pumps (31.0%), a milk dump line (29.9%), variable speed vacuum drives (26.9%), electronic milk meters (23.6%), automatic drafting (22.0%), automatic cluster flush (ACF) (15.2%), automatic teat sprayers (14.9%), and backing gates in the collecting yard (13.3%). The least common parlour add-ons included automatic identification systems (10.9%), dual vacuum systems (6.0%), non-electronic milk meters (5.7%), automatic mastitis detection systems (4.1%), and automatic in-cluster dipping (1.9%).

**Table 3:** Average number of technology add-ons per parlour by herd size quartiles

Quartiles	Average number of technology add-ons
1 (< 73 cows)	3.0
2 (73–105 cows)	4.6
3 (106–159 cows)	5.7
4 (> 159 cows)	7.0
**Average**	**5.1**

Cluster disinfection occurred in 34.4% of herds. Of these, 59.1% conducted manual disinfection whilst 40.9% used an automated system of cluster disinfection. When manually done, 53.3% conducted it after every high cell count cow, 44.0% conducted it after every cow milked in the parlour and 2.7% did it “at the end of milking” or for “cows that recently had mastitis” or “during treatment of cows with mastitis”. The most commonly used cluster disinfection agents were peracetic acid-based products regardless of method of disinfection. Cluster disinfection products were in concentrate form that required addition to water and mixing before use on farm in 56.7% of respondent cases and ready-to-use products on 37.8% of respondent farms.

#### Milking management

Fore-milking was conducted in cases where clots were identified in the milk filter in the previous milking (56.6%), for cows that were freshly calved (46.1%), for cows showing signs of clinical mastitis (hot, swollen and painful udders) (41.6%), and when increases in bulk tank SCC occurred (36.5%). In addition, fore-milking was conducted by 13.9% of farmers at every milking, 8.0% reported they never do it and 7.5% reported to do it at every morning milking only. ‘Other’ responses included “after receiving milk recording results”, “every morning during housing only”, “once per week” and “[if] udder rub [is] abnormal” (‘udder rub’ referring to a practice whereby farmers gently massage the udder prior to milking to check for any palpable abnormalities and to stimulate milk let-down).

When asked where they strip the foremilk, 79.6% stripped it onto the floor, 14.9% into a gloved hand, 2.7% into a strip cup and 0.9% into a bare, non-gloved hand. CMT testing was utilised on 56.9% of respondent farms.

In terms of pre-milking preparation, 32.1% of respondents reported not doing any udder preparation. A dry wipe was carried out for cleaning the external surface of the teat prior to milking on 37.5% of respondent farms, a wash with a hose on 10.2%, individual udder wash cloths (defined as one cloth used per cow) on 2.7% and a communal wash cloth (defined as use of a single wash cloth on more than one cow) on 1.6% of farms. Less frequent methods of washing included the use of an udder brush (1.3%) and a pre-milking wash cup (1.1%). Pre-spraying was implemented on 18.9% of farms and pre-dipping on 7.0% of farms. Drying was conducted using an individual (one piece per cow) dry cloth or piece of paper towel on 10.8% of farms and using a communal dry cloth or piece of paper towel (one piece used on more than one cow) on 6.5% of respondent farms. Other options for pre-milking udder preparation included “dry wipe [in] winter only”, “check for dirt and clean with hand”, “none except soiled teats washed” and “pre-spray high SCC cows” or “pre-spray in springtime”.

Post-milking teat disinfection most commonly involved spraying with a disinfectant solution, with 88.4% of farms using this method. Post-milking dipping occurred on 6.5% of farms and automatic in-cluster dipping on 0.5% of farms. No post-milking teat disinfection occurred on 3.0% of farms. ‘Other’ options (1.6%) included “post-spray in spring and autumn” and “spraying cows only when housed”. The most commonly used teat disinfection products contained chlorhexidine or lactic acid as their primary disinfectant agent; occasionally utilising a combination of the two with varying concentrations depending on brand.

Other udder hygiene practices involved clipping udders (18.8%), flaming udders (14.7%) and clipping tails (96.5%) in order to achieve better maintenance and ease of udder cleaning.

Gloves were always worn in the parlour on 81.6% of respondent farms. Gloves were sometimes worn in the parlour in 12.6% and never worn in 5.9% of responses. The most common type of gloves used were disposable gloves (85.2%), followed by reusable and washable gloves (8.1%).

#### SCC control

Mastitis treatment records were kept by 90.7% of respondents. Of these, 49.0% used a whiteboard, 48.7% used a farm book and 50.7% used a software application. 10.1% of respondents used another means of keeping mastitis treatment records. Given the nature of the checkbox question, participants could select more than one answer and therefore percentage figures equate to greater than 100%.

Collecting milk samples for bacteriological analysis was conducted rarely on 23.5% of respondent farms and never done on 15.5%. Reasons for doing it included the advice of a veterinarian (as opposed to being done autonomously) in 22.1% of cases, for both clinical and subclinical mastitis cases (22.4%), for clinical mastitis cases only (9.6%) and for subclinical mastitis cases only (6.9%). Advice on how to manage SCC was acquired from veterinarians by 76.9% of farmers, followed by discussion with colleagues (50.0%), processor milk quality advisors (39.2%) and websites (20.7%). Less frequently used sources of information on SCC management included other advisors (15.3%), magazines (14.5%), mastitis handbooks (11.6%) and on-site visits by mastitis experts (6.1%). Other sources of advice (2.4%) included “milk recording results” and “milking machine technician[s]”. Again, given the nature of the checkbox question, participants could select more than one answer and therefore percentage figures equate to greater than 100%. The practicality of milk recording reports or text alerts for managing SCC was rated at a ‘4’ or ‘5’ of the five-point scale by 90.8% of the survey respondents, where ‘5’ is a strong agreement to their usefulness.

When asked to choose what they considered most responsible for causing high SCC on farm, the most common answers were the milking parlour (33.2%), older cows in the herd (24.7%), housing conditions (16.6%) and the milking machine (9.9%). ‘Milking parlour’ refers to the general milking environment and associated milking management practices (e.g. parlour cleanliness, liner condition, pre- and post- milking practices, etc.), whereas ‘milking machine’ refers to machine-specific functions which may influence source and spread of mastitis (e.g. vacuum and pulsation settings, cluster detachment timings, etc.). Less frequently chosen answers included freshly calved cows (1.6%) and grassland (0.3%). ‘Other’ answers (7.2%) included “general weakness for health”, “not milking the cows yourself”, “slow action by the farmer to a problem cow which lets the issue spread” and “selective dry cow therapy”. The source of high SCC was deemed unknown by 6.4% of respondents. In terms of managing high SCC cows, 78.3% of respondents milk them alongside the rest of the herd whilst 16.3% milk them after the rest of the herd.

#### Farmer demographics

Respondents were 96.8% male and 3.2% female. The most common age range was 45–54 (34.0%) followed by 35–44 (25.3%) and 55–64 (24.5%). Remaining groups included 10.9% aged between 25 and 34, 4.8% aged 65 + and 0.5% in the younger range of 18–24 years of age. Respondents were most commonly dairying for 20–30 years (26.6%), with 19.2% being in dairy for greater than 40 years and 5.6% for less than five years. Levels of education varied from primary (1.6%), to secondary (9.1%), to third level (23.0%) to various levels of courses completed. These included certificate courses in agriculture such as the ‘Green Cert’ (35.9%) and other courses in agricultural colleges that lasted for one year (12.9%) or greater than one year (12.6%).

For 2021, 40.7% respondents were very happy (score of ‘8’, ‘9’ or ‘10’ on the 10-point satisfaction scale) with their SCC management whilst 17.3% were very worried about it (score of ‘1’, ‘2’ or ‘3’ on the 10-point satisfaction scale). For 2022, 48.3% were very happy with their SCC management and 15.2% were worried. High levels of confidence that a low SCC (< 200,000 cells/ml) is achievable on their farm was reported by 77.0% of respondents.

The effect of legislation changes on strict implementation of selective dry cow therapy was not predicted to affect the current drying-off practices of 34.2% of respondents (score of ‘8’, ‘9’ or ‘10’ on a 10-point scale), but was anticipated to greatly affect the practices of 22.5% of respondents (‘1’, ‘2’ or ‘3’ on a 10-point scale), based on their own responses. Confidence in the ability of selective dry cow therapy (SDCT) to control SCC was high (score of ‘8’, ‘9’ or ‘10’ on a 10-point scale) for 38.7% of respondents and low (‘1’, ‘2’ or ‘3’ on a 10-point scale) for 28.1% of respondents. For farmers already implementing SDCT, dry-off treatment with teat sealant alone was implemented based on individual cow SCC records on 62.9% of farms, on available records of clinical cases and their outcomes throughout the lactation for 54.5% of farms, on individual cow factors for 28.4%, on milk yield records for 22.2%, and on the results of individual CMT testing on 16.5% of farms. None of the above were used in decision making for 8.4% of farms. The most common SCC cut-off point for using teat sealant only dry-off treatments was 100,000 cells/ml (86.1%).

Satisfaction with their career in dairy was scored highly (‘8’, ‘9’ or ‘10’ on a 10-point scale) by 73.4% of respondents and less favourably (‘1’, ‘2’ or ‘3’) by 5.4% of survey respondents. The most common answer was a score of ‘10’ with 29.4% of respondents claiming immense fulfilment from their profession.

## Discussion

A study by Palma Molina et al. [23] documented the prevalence of certain technologies but did not examine milk quality on farms. Other Irish studies document certain aspects of management practices and their relationship with BTSCC [24, 35, 36], but did not tie these together with parlour technologies. This study documented the farm and parlour management practices, parlour technologies, milking management practices, SCC control strategies and farmer demographics on a sample of commercial Irish dairy farms, thereby filling current gaps in the literature. The geographical distribution of responses in this study aligns with the distribution of milk producers in Ireland. Most responses were in Munster (59.0%), particularly in Cork, the county with the greatest number of dairy cows according to the Central Statistics Office (CSO) [11]. According to the 2022 Teagasc National Farm survey [37], 72% of Irish dairy animals were in the south of the country. The national average herd size in 2022 was 93 cows [37], meaning our study has an inherent inference bias towards larger herds (average herd size of 131 cows). Herd size quartiles in this study reflect a smaller average herd size per quartile than other studies, such as those reported by Hogan et al. [38]. Cyclical, seasonal trends of BTSCC found in this study are similar to those reported by O’Connell et al. [34] for both calving patterns and herd size quartiles.

### Parlour technologies and management

Good facilities and modernization of dairy operations have been shown in the literature to correlate with improved production, efficiency and personal satisfaction [39]. In line with this, dairy farmers worldwide are becoming increasingly interested in the implementation of technology to replace labour and to improve efficiency [40–42]. Our study found that 46.8% of dairy parlours were less than 10 years of age, with 20.1% being less than five years of age. This could reflect growing dairy cow numbers on these particular farms which therefore necessitated increased space to accommodate them, or could be indicative of a need for modernization to suit current milking efficiency or labour demands. Despite this finding, 63.2% of respondents reported not implementing any major upgrades in the last five years. Though we can assume the new parlours fall under the 36.8% who did, we cannot verify this. Our study found that smaller herds had lower levels of technology add-ons (except for dual vacuum systems and automatic in-cluster dipping; Supplementary Table 4). The use of automation increased with herd size quartile (Table 3), for example herds in Q4 had an average of 7.0 technology add-ons compared to an average of 3.0 for Q1 herds (Table 3). However, similar levels of uptake of dual vacuum systems, automatic teat sprayers and non-electronic milk meters was noted across all four quartiles, as was increased implementation of automatic in-cluster dipping in lower quartile (Q1 and Q2) herds compared to no uptake in Q3 and Q4 respondent herds. The most common technological add-ons on respondent farms were in-parlour feeding systems (84.5%), automatic washers on the bulk tank (72.8%), ACRs (57.9%), and entrance or exit gates controlled from the pit of the parlour (52.2%). In this study, AMS parlours and rotaries had more parlour technologies on average than other types of parlour, with an average of 12.3 and 10.8 additions respectively (Supplementary Table 5). This finding was in agreement with the work of Yang et al. [43] which found that New Zealand rotary parlours were associated with a significantly increased number of automated labour-saving and data-capture technologies compared to herringbones. A recent study by Prendergast et al. [44] demonstrated that the number of automations on herringbone farms had a strong positive correlation with milking efficiency in terms of cow throughput and litres of milk harvested per hour, but a negligible correlation was documented between milking efficiency and automations for rotary parlours; likely due to minimal variation in automation presence on these farms. Our study also supports the Yang et al. [43] findings that ACRs and in-parlour feeding were among the most prevalent technological additions in both herringbones (34.9–66.3% and 53–78.3% respectively depending on the type of herringbone) and rotaries (100% for both respectively), though those in the Yang et al. [43] study had a much higher rate of automatic teat sprayer use than we found.

Another parlour management finding in this study was that 34.4% of survey respondents reported changing their liners only once per year. The current advice for spring-calving herds is to change liners every 2,000 milkings or every 6 months, whichever comes first [10]. This is true particularly for commonly encountered nitrile rubber liners, though silicone liners have been described in literature as having greater longevity of 5000 milkings or more [45, 46]. The liner is the only element of the milking machine that is in direct contact with the cow’s teat, therefore it is important that they are operating effectively. When liners are worn, they lose their shape and do not massage the teat correctly, resulting in longer milking times and reduced yields [47]. Worn liners are also a source of bacteria, particularly contagious mastitis-causing strains, as the cracks in the liner surface provide ideal environments for bacterial accumulation and growth [48].

### Milking management

Hygiene and stringent management practices during milking are linked with reduced farm-level SCC [24, 26, 34, 49, 50]. Pre-milking routines generally consist of stimulating milk let-down and preparing teats for cluster attachment. Regulation (EC) No 853/2004 states that raw milk should only be collected from cows which “present no sign of disease that might result in the contamination of milk… or a recognisable inflammation of the udder” [51]. Our study found that 13.9% of respondent farms conducted fore-milking as part of their daily milking routine and 8.0% reportedly never conduct it. This low prevalence of consistent fore-milking may inhibit farmers’ ability to identify any visible abnormalities in the milk such as blood or clots, which indicate evidence of clinical mastitis (CM) [52], especially given the low incidence of automatic mastitis detection systems (4.1%). Similarly, fore-milking is considered an effective stimulus for milk let-down [53]. Other pre-milking management practices include washing and drying of teats prior to cluster attachment. Clusters must be attached to clean, dry teats in order to minimise risk of environmental mastitis. However, we found that 32.2% of respondent farmers do not do any form of pre-milking teat preparation. Washing udders prior to milking, conducted by 14.5% of respondent farmers using either a hose or a wash cloth, is generally discouraged in the absence of adequate drying afterwards as pathogens can be distributed all over the surface of the udder and teats [30]. Should washing be required, only the teats should be washed, using minimal water, and should be thoroughly dried afterwards [52]. Galton et al. [54] demonstrated that washing and subsequent drying of teats resulted in low bacterial counts on teat ends and therefore less risk of new mastitis infection. Pankey et al. [55] showed that inadequate cleaning and drying of teats caused environmental mastitis pathogen numbers in milk to increase. Similarly, drying of teats via communal means, such as was reported by 6.5% of survey respondents, is strongly discouraged due to risk of mastitis pathogen spread from cow to cow and was associated with a higher monthly rate of clinical mastitis in a study by Ruegg [56]. Post-milking teat disinfection is required to destroy pathogens on teat skin at the end of milking. Only 3.0% of respondent farms reported no post-milking teat disinfection which, though worrying, were considerably outweighed by those conducting spraying (88.4%) and dipping (6.5%) of teats. Spraying offers an advantage in terms of time saving and more limited handling of cows that are particularly restless in the parlour or are first-time milkers [57]. Appropriate coverage is essential [58], with AHI Cell Check guidelines [59] suggesting a minimum of 10 ml of dip or 15 ml of spray per cow, per milking.

### SCC control

SCC and mastitis management practices were also investigated in this study. Almost six percent (5.9%) of farmers reported never wearing gloves in the parlour for milking, despite evidence in literature that glove-wearing practices are associated with reduced risk of spreading mastitis-causing bacteria from cow to cow via milkers’ hands [60, 61]. Persistently high SCC and recurrent incidence of CM were two of the greatest indicators for culling in this study. Lack of consistent fore-milking and also lack of engagement with bacteriology of milk samples, conducted ‘rarely’ or ‘never’ on 39.0% of respondent farms, could contribute to increased BTSCC through slower identification of clinical cases. A well-managed combination of hygiene practices and suitable antibiotic administration has long been proven to provide superior mastitis control to implementation of either measure alone [29]. Though the majority (77.0%) of respondents in our study strongly agreed that they believe a low SCC is attainable on their farms, 51.7% reported that amendments to the legislation surrounding SDCT will change their current management practices around drying-off and 28.1% reported concurringly low levels of confidence in their ability to maintain a low SCC in the advent of said changes. These legislative changes refer to the new Veterinary Medicines Regulation EU 2019/6 [62] which came into effect in January 2022. This legislation specifically prohibits prophylactic antibiotic use, other than in well-defined cases for an individual or a restricted number of animals when the risk of infection is very high and the consequences are likely to be severe (Article 107 [3]). National and farm-level recommendations “in support of improved mastitis control and intramammary antimicrobial stewardship in the Irish dairy industry” have been created by AHI CellCheck [28]. Farmers in our study found that support offered by milk recording reports and texts were useful, as were interactions with their veterinary professionals and milk quality advisors. This is an important finding, as fostering strong working relationships between farmers and their veterinary professionals, in particular, is more important than ever in order to ensure good antimicrobial stewardship and maintenance of optimal herd health. A novel approach to designing behaviour change interventions for antimicrobial use in Irish agriculture is described by Regan et al. [63]. Further factors which can influence behaviour in relation to antimicrobial use are outlined in an international study by McKernan et al. [64].

### Farmer demographics

General attitudes assessed in this study showed that farmers were generally quite content with their career in dairy. We found that 29.4% of respondents claimed immense fulfilment from their profession; awarding their love for their career a score of ‘10’ out of ‘10’ on a satisfaction scale. However, other studies have shown that there are considerable concerns in the dairy sector surrounding recruitment and retention of labour [65]. The intense seasonal workload associated with pasture-based farming [18, 38] can lead to increased levels of stress [66], mental health problems [67] and difficulties in sustaining an adequate quality of life [68]. Our study found that 63.3% of survey respondents were over the age of 45 and 66.1% of respondents had sustained a career in dairy for over 20 years, with 19.2% of these having farmed for over 40 years. Greater emphasis on incentives such as a good work-life balance and increased family time [69], and creation of a more sustainable workload are essential in enticing the younger generation towards a career in farming [70]. This supports recent research carried out by Huey et al. [71] and Regan et al. [72]. Moving forward, further efforts in offering farmers support and decision-making tools for farm and parlour management, as well as continued education for the professionals they most engage with to ensure there is consistency in advice and methods of advice dissemination, could be of great benefit to the Irish dairy industry.

### Study limitations

We are aware of a level of bias pertaining to the fact that respondent herd sizes are larger than the national average. It is important to note that this study was based on self-reported management practices, behaviour and attitudes of farmers. The farmers participating in this study could be different than the average Irish farmer because of their willingness to self-select participation in the survey (inferring a certain level of selection bias). There is also a chance that farmers reported socially desirable answers which could directly add bias to the results. It is important to note that, although the survey was extensive and developed alongside experts in the field of mastitis, the total dataset of parlour technologies, management practices, attitudes and behaviour regarding milking management may be incomplete.

## Conclusion

In this study, we successfully filled current knowledge gaps around farm management practices, parlour management including rates of technology adoption, general milking management, SCC control strategies and farmer attitudes and behaviour towards certain SCC management practices by completing a survey of Irish dairy farms. Our study found that the most common parlour technology additions were in-parlour feeders (84.5%), automatic washers on the bulk tank (72.8%) and ACRs (57.9%). Rotary parlours and AMS parlours had the most parlour technologies of any parlour type (10.8 and 12.3 add-ons on average respectively). Technology add-ons were less prevalent in smaller herds (average number for Q1 being 3.0 add-ons per herd compared to 7.0 for Q4 herds), except for dual vacuum systems and automatic in-cluster dipping. Despite finding areas for improvement with regard to frequency of liner changes (51.5% changing at the recommended intervals of every 2000 milkings or every six months), glove-wearing practices (18.5% wearing them sometimes or never), using a communal means of drying teats in the parlour (6.5% using a communal cloth or paper towel to dry teats prior to milking), and engagement with bacteriology of milk samples (rarely or never done on 39.0% of farms), we also found evidence of high levels of good management practices, including the documentation of mastitis treatment records (90.7%) and high use of post-milking teat disinfection (94.9% either dipping or spraying). We discovered that Irish dairy farmers are relatively content in their careers but face pressures following changes to the legislation regarding antimicrobial use in their herds. However, there are high levels of confidence that low herd level SCC is achievable on respondent farms. Farmers in our study found that support offered by milk recording reports and texts were useful for reducing their BTSCC, as were interactions with their veterinary professionals and milk quality advisors.

### Supplementary Information

**Supplementary Material 1.**

**Supplementary Material 2.**

## Data Availability

The participants of this study did not give written consent for their data to be shared publicly, so due to the sensitive nature of the research supporting data is not available.

## Abbreviations

SCC: Somatic cell count
AMS: Automatic milking system
BTSCC: Bulk tank somatic cell count
ACR: Automatic cluster remover
PLF: Precision livestock technology
CMT: California Mastitis Test
AHI: Animal Health Ireland
ICBF: Irish Cattle Breeding Federation
ACF: Automatic cluster flush
SDCT: Selective dry cow therapy
CSO: Central Statistics Office
CM: Clinical mastitis
