# Supplementary material for: Survey of farm, parlour and milking management, parlour technologies, SCC control strategies and farmer demographics on Irish dairy farms
Source: Ir Vet J. 2024 May 6;77:8. doi: 10.1186/s13620-024-00267-y (PMC11071209; doi:10.1186/s13620-024-00267-y)
Supplement: Supplementary file 1 — Supplementary Material 1. [file 13620_2024_267_MOESM1_ESM.docx]

# **Supplementary Materials**

## **Supplementary Materials 1: Survey Design (n=376)**

### **Questions: Consent**

| **Question number** | **Question** | **Type of question** | **Number of total responses** |
| --- | --- | --- | --- |
| 1 | I am fully informed and consent to my data from this survey and from ICBF being used in this study | Checkbox (required an answer for continuation) | 376 |
| 2 | I consent to being contacted with regard to participation in the on-farm study being conducted in late 2022 / early 2023 | Checkbox (required an answer for continuation) | 361 |

### **Questions: Contact information**

| **Question number** | **Question (n = respondent answers)** | **Type of question** | **Answers** | **%** | **n** |
| --- | --- | --- | --- | --- | --- |
| 1 | Contact information: | Textboxes (contact information format provided by SurveyMonkey) | Name  Address  Eircode  Email address  Phone number  *Missing data ^1^* |  | -  -  -  -  -  - |
|  |  |  |  |  |  |
| 2 | Herd number of the **main milking herd** | Textbox | *Missing data* |  | *-* |
| 3 | Does this herd milk in more than one parlour over the course of lactation? E.g. summer block parlour | Multiple choice | Yes  No  *Missing data* | 3.7  96.3 | 14  362  - |
| 4 | Is this farm managed as a partnership? | Multiple choice | Yes  No  *Missing data* | 22.3  77.7 | 84  292  - |

### **Questions: Farm-specific information**

| **Question number** | **Question** | **Type of question** | **Answers** | **%** | **n** |
| --- | --- | --- | --- | --- | --- |
| 1 | Parlour type  Please answer for the parlour that accounts for the majority of milkings per year | Multiple choice  Other: textbox | Swing-over herringbone  Double-up herringbone  Herringbone with recording jars  Parallel / Side-by-side  Midline herringbone  Rotary  Robot / Automated Milking System (AMS)  Other (please specify)  *Missing data* | 59.1  4.8  22.2  6.7  -  2.7  2.7  1.9 | 221  18  83  25  -  10  10  7  *2* |
| 2 | Parlour manufacturer:  Please answer for the parlour that accounts for the majority of milkings per year | Dropdown | ATL Agricultural Technology Ltd  BouMatic  Dairymaster  DeLaval  ElectroMech Agri  Fullwood Packo  Gascoigne  G.E.A.  Lely  Milfos  Pearson  Waikato  Hybrid (components from more than one manufacturer)  Other  *Missing data* | 1.4  3.2  31.9  33.8  -  6.2  8.6  1.6  2.4  -  3.2  0.8  4.1  2.7 | 5  12  118  125  -  23  32  6  9  -  12  3  15  10  *6* |
| 3 | Do you keep mastitis treatment records?  (If yes, go to Q4)  (If no, go to Q5) | Multiple choice | Yes  No  *Missing data* | 90.7  9.3 | 341  35  - |
| 4 | If you keep mastitis treatment records, how are they kept?  Please select one or all that apply to your farm | Checkboxes | Whiteboard  Farm recording book  App  Other  *Missing data* | 49.0  48.7  50.7  10.1 | 150  149  155  31  35 |
| 5 | Number of milking cows in **2022** (ICBF average), e.g. 80:  Please put only a number in the box | Textbox | *Missing data* |  | 38 |
| 6 | Number of milking cows in **2021 (last year)** (ICBF average), e.g. 80:  Please put only a number in the box | Textbox | *Missing data* |  | 5 |
| 7 | Number of milking units, e.g. 20: Please put only a number in the box | Textbox | *Missing data* |  | 4 |
| 8 | Number of cows culled in the last 12 months specifically for persistent mastitis / high cell count, e.g. 25:  Please put only a number in the box | Textbox | *Missing data* |  | 5 |
| 9 | Frequency of milking | Multiple choice  Other: textbox | Once per day  Twice per day  Voluntary milking / AMS system  Other (please specify)  *Missing data* | 1.3  95.2  2.7  0.8 | 5  357  10  3  1 |

### **Questions: Parlour-specific information**

| **Question number** | **Question** | **Type of question** | **Answers** | **%** | **n** |
| --- | --- | --- | --- | --- | --- |
| 1 | Normal **morning** milking time and average duration (Duration = Time from milking machine turned on until cleaning is complete): Please put only numbers in the box | Textbox | Normal morning milking start time (**AM**) e.g. 7:30 or 7 30  *Missing data*  Average duration of morning milking (**hours and minutes**) e.g. 1 hours 30 minutes – 1.30 or 1 30  *Missing data* |  | 10  35 |
| 2 | Normal **evening** milking time and average duration (Duration = Time from milking machine turned on until cleaning is complete): Please put only numbers in the box | Textbox | Normal morning milking start time (**PM**) e.g. 5:30 or 5 30  *Missing data*  Average duration of morning milking (**hours and minutes**) e.g. 1 hours 30 minutes – 1.30 or 1 30  *Missing data* |  | 18  41 |
| 3 | How many people are in the parlour for milking during peak lactation? If people come and go during milking, count these as a 0.5; e.g. 2.5: Please put only a number in the box | Textbox | Morning milking  *Missing data*  Evening milking  *Missing data* |  | 39  24 |
| 4 | Who are the ‘milkers’ on the farm? Please select one or all that apply to your farm | Checkboxes  Other: textbox | Myself  Family  Employee  Other (please specify)  *Missing data* | 90.7  57.1  33.6  6.0 | 332  209  123  22  10 |
| 5 | Age of milking system since installation on your farm (**years**); e.g. 4 (i.e. when the milking system was purchased – not age since last upgrade):  Please put only a number in the box | Textbox | <5  5-10  11-15  16-20  21-25  26-30  31-35  36-40  41-45  46-50  Other  *Missing data* | 20.1  26.7  16.3  11.2  9.4  7.5  2.4  2.4  2.4  1.3  0.3 | 75  100  61  42  35  28  9  9  9  5  1  2 |
| 6 | Have you implemented any major upgrades to your milking system in the last **5** years? E.g. bailing system, ACRs, etc.. | Multiple choice  If yes: textbox | Yes  No  *Missing data*  If yes, what upgrades have you installed? | 36.8  63.2 | 135  232  9  - |
| 7 | How many times **per year** is the milking machine serviced? | Multiple choice | Less than once per year  Once per year  Twice per year  More than twice per year  *Missing data* | 8.0  72.1  15.2  4.8 | 30  271  57  18  - |
| 8 | Milk line: Where is your milk line situated in the parlour? | Multiple choice | Low level (below where the cows are standing)  Mid level (at or above where the cows are standing)  AMS / Robot parlour  *Missing data* | 7.2  90.1  2.7 | 27  337  10  2 |
| 9 | Pulsation type: Please select from the following options which pulsation type you have on your farm | Multiple choice  Other: textbox | Simultaneous (4x0): Pressure is applied to, and released from, all teats at the same time  Alternating (2x2): Pressure is applied to two teats while the other two rest and then switches over  One pulsator per teat: Pressure is applied to, and released from, one teat at a time  Other (please specify)  *Missing data* | 41.3  53.9  3.8  1.1 | 154  201  14  4  3 |
| 10 | Which, if any, of the following do you employ to assist with cow positioning in the parlour?  Please select from the following options those which you have on your farm (tick *all* boxes that apply) | Checkbox | Manual bailing system  Sequential bailing system  Individual mangers  Zig-zag rump rail  Straight breast rail  Adjustable breast rail  Straight rump rail  Other  *Missing data* | 13.7  5.5  59.6  14.8  7.1  8.5  39.3  1.4 | 50  20  217  54  26  31  143  5  12 |
| 11 | Parlour add-ons: Please select from the following list which parlour add-ons you have on your farm (tick *all* of the boxes that apply) | Checkbox | Automatic cluster removers  Dual vacuum or similar system for pulsation (alternatives to ACRs)  Automatic cluster flush  Automatic in-cluster dipping  Automatic washer on milking machine  Automatic washer on bulk tank  In-parlour feeding  Automatic cow ID system  Automatic teat sprayers  Electronic milk meters  Non-electronic milk meters  Milk diversion line / Dump line  Automatic mastitis detection  Automatic drafting system  Backing gate in collecting yard  Variable speed vacuum pump  Variable speed milk pump  Entrance / Exit gates controlled from the pit  No technology add-ons  *Missing data* | 57.9  6.0  15.2  1.9  34.8  72.8  84.5  10.9  14.9  23.6  5.7  29.9  4.1  22.0  13.3  26.9  31.0  52.2  4.9 | 213  22  56  7  128  268  311  40  55  87  21  110  15  81  49  99  114  192  18  8 |
| 12 | Frequency of liner changes | Multiple choice  Other: textbox | Once per year  Twice per year  Three times per year  Every 2 years  Every 2000 milkings  Every 2500 milkings  Other (please specify)  *Missing data* | 34.4  44.8  4.5  1.3  6.7  6.1  2.1 | 129  168  17  5  25  23  8  1 |
| 13 | Do you employ cluster disinfection on your farm?  (If yes, go to Q14)  (If no, go to cow-specific information Q1) | Multiple choice | Yes  No  *Missing data* | 34.4  65.6 | 129  246  1 |
| 14 | If yes, is cluster disinfection manual or automatic?  (If manual, go to Q15)  (If automatic, go to Q16) | Multiple choice | Manual  Automatic  *Missing data* | 59.1  40.9 | 75  52  2 |
| 15 | If manual cluster disinfection, when are clusters disinfected? | Multiple choice  Other: textbox | After every cow that has been milked  After every high cell count cow that has been milked  Other (please specify)  *Missing data* | 44  53.3  2.7 | 33  40  2  - |
| 16 | Product(s) used for cluster disinfection  Please specify brand name if possible | Textbox | *Missing data* |  | 262 |
| 17 | Product(s) used for cluster disinfection – concentrate or pre-mixed? | Multiple choice | Concentrate / ‘Make it yourself’  Pre-mixed / ‘Ready to go’  Other  *Missing data* | 56.7  37.8  5.5 | 72  48  7  2 |

### **Questions: Cow-specific information**

| **Question number** | **Question** | **Type of question** | **Answers** | **%** | **n** |
| --- | --- | --- | --- | --- | --- |
| 1 | When, if ever, do you employ strip milking / fore-milking on your farm?:  Please select all that apply to your farm | Checkbox  Other: textbox | I never employ fore-milking on my farm  Every milking  Every morning milking only  When clinical cases of mastitis are identified in individual cows in the parlour  Immediately after calving  When clots are identified in the milk filter  When there is an increase in bulk tank SCC  Other (please specify)  *Missing data* | 8.0  13.9  7.5  41.6  46.1  56.6  36.5  2.9 | 30  52  28  155  172  211  136  11  3 |
| 2 | If fore-milking is done, it is __? | Multiple choice  Other: textbox | Onto the floor  Into a strip cup  Into an ungloved / bare hand  Into a gloved hand  Other (please specify)  *Missing data* | 79.6  2.7  0.9  14.9  1.8 | 261  9  3  49  6  48 |
| 3 | Do you conduct CMT testing on your farm to identify high SCC cows? | Multiple choice | Yes  No  *Missing data* | 56.9  43.1 | 211  160  5 |
| 4 | Pre-milking udder preparation: Please select from the following practices that reflect the **pre-milking** stage of the milking process on your farm (tick *all* of the boxes that apply) | Checkbox  Other: textbox | None  Dry wipe  Hose  Pre-dipping  Pre-spraying  Washing with an individual wet udder cloth (one cloth used per cow)  Washing with a communal wet udder cloth (one cloth used on more than one cow)  Pre-milking wash cup (AMS)  Udder brush  Drying with an individual udder cloth / paper towel (one cloth used per cow)  Drying with a communal udder cloth / paper towel (one cloth used on more than one cow)  Other (please specify)  *Missing data* | 32.1  37.5  10.2  7.0  18.9  2.7  1.6  1.1  1.3  10.8  6.5  7.0 | 119  139  38  26  70  10  6  4  5  40  24  26  5 |
| 5 | Post-milking teat disinfection: Please select from the following the practices that reflect the **post-milking** stage of the milking process on your farm | Multiple choice  Other: textbox | None  Spraying  Dipping  Automatic in-cluster dipping  Other (please specify)  *Missing data* | 3.0  88.4  6.5  0.5  1.6 | 11  327  24  2  6  *6* |
| 6 | Teat disinfection products | Textbox | Product(s) used for **pre-milking**  *Missing data*  Product(s) used for **post-milking**  *Missing data*  Average volume used per month (estimate in **litres**), e.g. 90  *Missing data* |  | 254  68  58 |
| 7 | Udder hygiene: Please select the options that apply to the udder hygiene practices on your farm (tick *all* of the boxes that apply) | Checkbox  Other: textbox | Clip udders  Flame udders  Clip tails  Other (please specify)  *Missing data* | 18.8  14.7  96.5  0.5 | 70  55  360  2  3 |
| 8 | Do you wear gloves in the parlour while milking? | Multiple choice | Always  Sometimes  Never  *Missing data* | 81.6  12.6  5.9 | 305  47  22  2 |
| 9 | Please choose the option that best describes your glove-wearing practices in the parlour during milking | Multiple choice  Other: textbox | Disposable rubber gloves  Reusable washable gloves  Bare hands  Other (please specify)  *Missing data* | 85.2  8.1  6.4  0.3 | 306  29  23  1  17 |
| 10 | What number of your **lactating** cows got teat sealant ONLY at drying-off in **2021 (last year)**? E.g. 90 – Teat sealant use in a number of lactating cows (not cull cows or heifers):  Please put only a number in the box | Textbox | *Missing data* |  | 14 |
| 11 | What number of your **lactating** cows were treated with intramammary antibiotics ONLY at drying-off in **2021 (last year)**?  E.g. 15 – Antibiotic use in a number of lactating cows (not cull cows or heifers):  Please put only a number in the box | Textbox | *Missing data* |  | 62 |
| 12 | What number of your **lactating** cows were treated with teat sealant AND intramammary antibiotics at drying-off in **2021 (last year)**?  E.g. 90 – Antibiotic and teat sealant combined use in a number of lactating cows (not cull cows or heifers):  Please put only a number in the box | Textbox | *Missing data* |  | 11 |
| 13 | Antibiotic product(s) used in **2021 (last year)** | Textbox | *Missing data* |  | 48 |
| 14 | Maximum number of **cows** dried-off **per person per day** during the **2021 season**? – Please specify the maximum number of cows that were dried-off by one person during the 2021 season:  Please put only a number in the box | Textbox | *Missing data* |  | 5 |
| 15 | How many of your **in-calf** **heifers** got teat sealed between **Autumn 2021** and **Spring 2022**? E.g. 20:  Please put only a number in the box | Textbox | *Missing data* |  | 9 |

### **Questions: Farmer-specific information**

| **Question number** | **Question** | **Type of question** | **Answers** | **%** | **n** |
| --- | --- | --- | --- | --- | --- |
| 1 | Gender | Multiple choice | Male  Female  Other  *Missing data* | 96.8  3.2  - | 363  12  -  1 |
| 2 | Age | Multiple choice | 18-24  25-34  35-44  45-54  55-64  65+  *Missing data* | 0.5  10.9  25.3  34.0  24.5  4.8 | 2  41  95  128  92  18  - |
| 3 | How many years have you spent dairying? | Multiple choice | <5  5-10  10-20  20-30  30-40  40+  *Missing data* | 5.6  13.3  14.9  26.6  20.3  19.2 | 21  50  56  100  76  72  1 |
| 4 | What is the highest level to which you have *completed* your education? | Multiple choice  Other: textbox | Primary school  Junior Certificate  Leaving Certificate  Certificate in Agriculture (e.g. Green Cert)  Agricultural college: One year  Agricultural college: More than one year  Third-level education: Undergraduate degree  Third-level education: Postgraduate degree  Prefer not to answer  Other (please specify)  *Missing data* | 1.6  4.0  5.1  35.9  12.9  12.6  16.1  5.9  0.8  5.1 | 6  15  19  134  48  47  60  22  3  19  3 |
| 5 | Have you availed of a free TASAH Dry Cow Consult? | Multiple choice | Yes  No  *Missing data* | 23.1  76.9 | 87  289  - |
| 6 | How often do you engage with bacteriology / culture and sensitivity of milk samples? | Matrix / Rating scale | For clinical mastitis only (cows with hot, swollen, painful udders with visible milk abnormalities)  For subclinical mastitis only (cows with a high SCC but normal udder and milk)  For both clinical and subclinical mastitis cows  Only when I am advised by the vet  Rarely  Never  *Missing data* | 9.6  6.9  22.4  22.1  23.5  15.5 | 36  26  84  83  88  58  1 |
| 7 | How did you feel about your overall SCC for **2021**?:  1=”very worried”, 5=”neutral”, 10=”very happy” | Matrix / Rating scale | 1  2  3  4  5  6  7  8  9  10  *Missing data* | 3.7  4.3  9.3  6.9  16.0  9.3  9.6  15.7  9.3  15.7 | 14  16  35  26  60  35  36  59  35  59  1 |
| 8 | How do you feel about your overall SCC for **2022** thus far?:  1=”very worried”, 5=”neutral”, 10=”very happy” | Matrix / Rating scale | 1  2  3  4  5  6  7  8  9  10  *Missing data* | 3.5  5.6  6.1  6.7  12.0  6.9  10.9  17.1  16.5  14.7 | 13  21  23  25  45  26  41  64  62  55  1 |
| 9 | I seek advice about cell count / SCC from…: Please select as many options as you personally use | Checkbox  Other: textbox | My vet  My co-op milk quality advisor  Other advisor  Discussion with colleagues / other farmers  Magazines  Websites  Mastitis handbooks  On-site visits by specialist mastitis management experts  Other (please specify)  *Missing data* | 76.9  39.2  15.3  50.0  14.5  20.7  11.6  6.1  2.4 | 286  146  57  186  54  77  43  23  9  4 |
| 10 | I find that milk recording reports / texts are useful for reducing SCC..  1=”Strongly disagree”, 5=”Strongly agree” | Multiple choice | Strongly agree  Agree  Neither agree nor disagree  Disagree  Strongly disagree  *Missing data* | 62.5  28.3  7.8  0.8  0.5 | 232  105  29  3  2  5 |
| 11 | I believe that a low SCC (<200,000 cells/ml) is achievable on my farm..  1=”Strongly disagree”, 5=”Strongly agree” | Multiple choice | Strongly agree  Agree  Neither agree nor disagree  Disagree  Strongly disagree  *Missing data* | 77.0  19.8  2.4  0.5  0.3 | 288  74  9  2  1  2 |
| 12 | I believe that a high SCC on farms comes **predominantly** from…: Please select one option that you feel is the most related to high SCC on farms in general | Multiple choice  Other: textbox | The milking machine  The milking process / milking practices  Heifers  Freshly calved cows  Older cows  Housing  Grassland  Unknown source  Other (please specify)  *Missing data* | 9.9  33.2  0  1.6  24.7  16.6  0.3  6.4  7.2 | 37  124  0  6  92  62  1  24  27  3 |
| 13 | How will the new legislation on antibiotic use affect your current antibiotic usage at dry-off? 1=”it will not affect it at all”, 5=”neutral”, 10=”it will drastically affect it” | Matrix / Rating scale | 1  2  3  4  5  6  7  8  9  10  *Missing data* | 11.9  4.9  5.7  4.6  21.3  8.6  8.9  14.8  6.2  13.2 | 44  18  21  17  79  32  33  55  23  49  5 |
| 14 | I am confident that I can manage my SCC with selective dry cow therapy..:  1=”not confident at all”, 5=”neutral”, 10=”very confident” | Matrix / Rating scale | 1  2  3  4  5  6  7  8  9  10  *Missing data* | 14.0  4.1  10.0  6.8  15.9  5.7  4.9  17.6  7.0  14.1 | 52  15  37  25  59  21  18  65  26  52  6 |
| 15 | If you are using **teat sealant alone** at drying-off in some of your cows, what information do you use to decide this treatment?: Please select as many that apply to you | Checkbox  Other: textbox | Records of clinical cases and their outcomes throughout the lactation  Milk yield records  Cow factors (age, teat condition, temperament)  CMT testing  Individual cow records  None of the above  Other (please specify)  *Missing data* | 54.5  22.2  28.4  16.5  62.9  8.4  3.9 | 182  74  95  55  210  28  13  42 |
| 16 | Where **teat seal only** is administered, what is the SCC threshold used to select cows? – Answer in thousand cells/ml, i.e. 300,000 cells/ml = 300:  Please type your answer for SCC cut-off into the box provided | Textbox | *Missing data* |  | 84 |
| 17 | I milk my high SCC cows.. | Multiple choice  Other: textbox | With the rest of the herd  Before the rest of the herd  After the rest of the herd  Other (please specify)  *Missing data* | 78.3  -  16.3  5.4 | 288  -  60  20  8 |
| 18 | On your farm, rank the following reasons from first to last (1-7) relating to your decisions to cull cows: Please rank the options relevant to practices on **your** farm.  1=”most frequent reason”, 7=”least frequent reason” | Ranking | Age   - 1 - 2 - 3 - 4 - 5 - 6 - 7   *Missing data*  Behaviour   - 1 - 2 - 3 - 4 - 5 - 6 - 7   *Missing data*  Fertility   - 1 - 2 - 3 - 4 - 5 - 6 - 7   *Missing data*  Lameness   - 1 - 2 - 3 - 4 - 5 - 6 - 7   *Missing data*  Poor milk production   - 1 - 2 - 3 - 4 - 5 - 6 - 7   *Missing data*  Recur CM   - 1 - 2 - 3 - 4 - 5 - 6 - 7   *Missing data*  High SCC   - 1 - 2 - 3 - 4 - 5 - 6 - 7   *Missing data* | 11.6  8.6  6.4  6.6  13.5  18.0  35.4  5.5  6.6  6.9  10.7  14.3  26.1  29.9  39.1  11.1  14.4  12.2  11.7  7.1  4.3  7.7  12.9  18.4  24.5  17.9  12.1  6.6  4.7  7.7  15.9  18.4  23.3  21.4  8.8  12.3  28.4  20.8  16.1  11.2  7.4  3.8  19.9  25.1  17.7  11.4  7.4  7.4  11.2 | 42  31  23  24  49  65  128  14  20  24  25  39  52  95  109  12  144  41  53  45  43  26  16  8  28  47  67  89  65  44  24  12  17  28  58  67  85  78  32  11  45  104  76  59  41  27  14  10  73  92  65  42  27  27  41  9 |
| 19 | On a scale of 1 to 10, I rate my love of being a dairy farmer at a…:  1 to 10 = low to high | Matrix / Rating scale | 1  2  3  4  5  6  7  8  9  10  *Missing data* | 1.6  1.6  2.2  1.3  4.9  3.8  11.3  25.1  18.9  29.4 | 6  6  8  5  18  14  42  93  70  109  5 |

^1^ ‘Missing data’ relates to unanswered questions. All percentage calculations are based on relative response rates to each question.
