# Supplementary material for: Survey of farm, parlour and milking management, parlour technologies, SCC control strategies and farmer demographics on Irish dairy farms
Source: Ir Vet J. 2024 May 6;77:8. doi: 10.1186/s13620-024-00267-y (PMC11071209; doi:10.1186/s13620-024-00267-y)
Supplement: Supplementary file 2 — Supplementary Material 2. [file 13620_2024_267_MOESM2_ESM.docx]

# **Supplementary Tables**

## **Supplementary Table 4: Types of technological parlour add-on count by herd size quartile**

|  |  |  | **Parlour technology add-ons** | | | | | | | | | | | | | | | | | |
| --- | --- | --- | --- | --- | --- | --- | --- | --- | --- | --- | --- | --- | --- | --- | --- | --- | --- | --- | --- | --- |
| **Quartiles** | **Number** | **NONE** | **ACR** | **DV** | **ACF** | **AICD** | **AWMM** | **AWBT** | **IPF** | **AID** | **ATS** | **EMM** | **NEMM** | **MDL** | **AMD** | **ADS** | **BGC** | **VSVP** | **VSMP** | **EGP** |
| 1 | 93 | 14 | 29 | 5 | 8 | 3 | 13 | 52 | 61 | 4 | 11 | 11 | 4 | 14 | 4 | 3 | 5 | 10 | 13 | 28 |
| 2 | 93 | 3 | 47 | 5 | 12 | 4 | 28 | 66 | 79 | 5 | 14 | 14 | 8 | 25 | 4 | 12 | 7 | 26 | 27 | 49 |
| 3 | 94 | 0 | 63 | 7 | 19 | 0 | 39 | 72 | 86 | 12 | 14 | 30 | 5 | 37 | 6 | 27 | 11 | 28 | 31 | 52 |
| 4 | 88 | 1 | 74 | 5 | 17 | 0 | 48 | 78 | 85 | 19 | 16 | 32 | 4 | 34 | 1 | 39 | 26 | 35 | 43 | 63 |
| **Total** | **368**^1^ | **18** | **213** | **22** | **56** | **7** | **128** | **268** | **311** | **40** | **55** | **87** | **21** | **110** | **15** | **81** | **49** | **99** | **114** | **192** |

Add-ons: ACR = Automatic cluster removers, DV = Dual vacuum or similar system for pulsation, ACF = Automatic cluster flush, AICD = Automatic in-cluster dipping, AWMM = Automatic washer on milking machine, AWBT = Automatic washer on bulk tank, IPF = In-parlour feeding, AID = Automatic ID system, ATS = Automatic teat sprayers, EMM = Electronic milk meters, NEMM = Non-electronic milk meters, MDL = Milk diversion or Dump line, AMD = Automatic mastitis detection, ADS = Automatic drafting system, BGC = Backing gates in the collecting yard, VSVP = Variable speed vacuum pump, VSMP = Variable speed milk pump, EGP = Entrance and exit gates controlled from pit

^1^ 8 herds removed due to unanswered question

## **Supplementary Table 5: Total number and mean parlour technological add-ons by parlour type**

|  |  | **Number of parlour technological add-ons** | | | |
| --- | --- | --- | --- | --- | --- |
| **Parlour Type** | **Number** | **Mean** | **Std** | **Min** | **Max** |
| Abreast | 1 | 0 | 0 | 0 | 0 |
| Automatic milking system | 9 | 12.3 | 2 | 9 | 15 |
| Double-up herringbone | 17 | 3.8 | 2.4 | 1 | 9 |
| Herringbone | 1 | 2 | 0 | 2 | 2 |
| Herringbone (no recording jars) | 2 | 1 | 1.4 | 0 | 2 |
| Herringbone (with recording jars) | 83 | 2.8 | 1.9 | 0 | 9 |
| Parallel | 25 | 3.9 | 3.4 | 0 | 11 |
| Parallel (with recording jars) | 1 | 3 | 0 | 3 | 3 |
| Rapid exit | 1 | 10 | 0 | 10 | 10 |
| Rotary | 10 | 10.8 | 3.2 | 4 | 14 |
| Swing-over herringbone | 216 | 5.7 | 3 | 0 | 12 |
| **Total** | **366^1^** |  |  |  |  |

^1^ Two herds removed due to unknown parlour type, 8 herds removed due to unanswered question

## **Supplementary Table 6: Parlour add-ons to assist with cow positioning by parlour type**

|  |  | **Cow-positioning parlour add-ons** | | | | | | | |
| --- | --- | --- | --- | --- | --- | --- | --- | --- | --- |
| **Parlour Type** | **Number** | **MB** | **SB** | **IM** | **ZZRR** | **SBR** | **ABR** | **STRR** | **OTHER** |
| Automatic milking system | 5 |  |  | 4 |  |  |  |  | 1 |
| Bucket plant | 1 |  |  | 1 |  |  |  |  |  |
| Double-up herringbone | 18 |  |  | 16 |  |  |  | 10 |  |
| Herringbone | 1 |  |  |  |  |  |  | 1 |  |
| Herringbone (no recording jars) | 1 |  |  | 1 |  |  |  |  |  |
| Herringbone (with recording jars) | 82 | 7 | 2 | 55 | 7 | 4 | 2 | 37 | 1 |
| Parallel | 24 | 13 | 4 | 6 | 1 | 2 |  | 10 | 1 |
| Parallel (with recording jars) | 1 | 1 |  | 1 |  |  |  | 1 |  |
| Rapid exit | 1 |  |  | 1 |  |  |  |  |  |
| Rotary | 8 |  |  | 7 |  |  |  | 1 | 1 |
| Swing-over herringbone | 220 | 29 | 14 | 123 | 45 | 20 | 29 | 82 | 1 |
| **Total** | **362 ^1^** | **50** | **20** | **215** | **53** | **26** | **31** | **142** | **5** |

Positioning add-ons: MB = Manual bailing system, SB = Sequential bailing system, IM = Individual mangers, ZZRR = Zig-zag rump rail, SBR = Straight breast rail, ABR = Adjustable breast rail, STRR = Straight rump rail

^1^ Two herds removed due to unknown parlour type, 12 herds removed due to unanswered question

## **Supplementary Table 7: Total number of parlour cow-positioning add-ons by parlour type**

|  |  | **Total cow-positioning parlour add-ons** | | | |
| --- | --- | --- | --- | --- | --- |
| **Parlour Type** | **Number** | **Mean** | **Std** | **Min** | **Max** |
| Automatic milking system | 5 | 1 | 0 | 1 | 1 |
| Bucket plant | 1 | 1 | 0 | 1 | 1 |
| Double-up herringbone | 18 | 1.4 | 0.5 | 1 | 2 |
| Herringbone | 1 | 1 | 0 | 1 | 1 |
| Herringbone (no recording jars) | 1 | 1 | 0 | 1 | 1 |
| Herringbone (with recording jars) | 82 | 1.4 | 0.6 | 1 | 3 |
| Parallel | 24 | 1.5 | 0.9 | 1 | 4 |
| Parallel (with recording jars) | 1 | 3 | 0 | 3 | 3 |
| Rapid exit | 1 | 1 | 0 | 1 | 1 |
| Rotary | 8 | 1.1 | 0.4 | 1 | 2 |
| Swing-over herringbone | 220 | 1.6 | 0.6 | 1 | 3 |
| **Total** | **362 ^1^** |  |  |  |  |

^1^ Two herds removed due to unknown parlour type, 12 herds removed due to unanswered question

|  |  |  |  |  |  |  |  |  |  |  |  |  |  |  |  |  |  |  |  |  |
| --- | --- | --- | --- | --- | --- | --- | --- | --- | --- | --- | --- | --- | --- | --- | --- | --- | --- | --- | --- | --- |
|  |  |  |  | **Parlour technological add-ons** | | | | | | | | | | | | | | | | |
| **Parlour Type** | **Number** | **None** | **ACR** | **DV** | **ACF** | **AICD** | **AWMM** | **AWBT** | **IPF** | **AID** | **ATS** | **EMM** | **NEMM** | **MDL** | **AMD** | **ADS** | **BGC** | **VSVP** | **VSMP** | **EGP** |
| AB | 1 | 1 |  |  |  |  |  |  |  |  |  |  |  |  |  |  |  |  |  |  |
| AMS | 9 |  | 7 | 3 | 9 | 5 | 8 | 9 | 9 | 9 | 9 | 8 | 1 | 7 | 9 | 8 |  | 6 | 1 | 3 |
| DUH | 17 |  | 10 | 1 | 1 |  | 3 | 11 | 15 | 1 |  | 3 | 1 | 5 |  | 1 |  | 3 | 2 | 7 |
| H | 1 |  |  |  |  |  |  | 1 | 1 |  |  |  |  |  |  |  |  |  |  |  |
| HNRJ | 2 | 1 |  |  |  |  |  |  | 1 |  | 1 |  |  |  |  |  |  |  |  |  |
| HRJ | 83 | 8 | 29 | 3 | 5 |  | 7 | 44 | 63 | 1 | 8 | 4 | 2 | 8 | 1 | 3 | 8 | 6 | 13 | 27 |
| PLL | 25 | 2 | 8 | 1 | 1 |  | 8 | 17 | 19 | 1 | 4 | 3 | 3 | 7 |  | 3 |  | 6 | 5 | 11 |
| PLLRJ | 1 |  | 1 |  |  |  |  | 1 | 1 |  |  |  |  |  |  |  |  |  |  |  |
| RE | 1 |  | 1 |  |  |  | 1 | 1 | 1 | 1 |  | 1 |  |  |  | 1 | 1 | 1 |  | 1 |
| R | 10 |  | 10 |  | 4 |  | 10 | 9 | 10 | 6 | 9 | 6 | 2 | 5 |  | 9 | 8 | 7 | 8 | 5 |
| SOH | 216 | 6 | 145 | 14 | 35 | 2 | 90 | 173 | 189 | 20 | 24 | 61 | 12 | 77 | 5 | 55 | 31 | 69 | 84 | 137 |
| **Total** | **366^1^** | **18** | **211** | **22** | **55** | **7** | **127** | **266** | **309** | **39** | **55** | **86** | **21** | **109** | **15** | **80** | **48** | **98** | **113** | **191** |

## **Supplementary Table 8: Technological parlour add-on numbers by parlour type**

Parlour types: AB = Abreast, AMS = Automatic milking system, DUH = Double-up herringbone, H = Herringbone, HNRJ = Herringbone with no recording jars, HRJ = Herringbone with recording jars, PLL = Parallel, PLLRJ = Parallel with recording jars, RE = Rapid exit, R = Rotary, SOH = Swing-over herringbone

Parlour technological add-ons: ACR = Automatic cluster removers, DV = Dual vacuum or similar system for pulsation, ACF = Automatic cluster flush, AICD = Automatic in-cluster dipping, AWMM = Automatic washer on milking machine, AWBT = Automatic washer on bulk tank, IPF = In-parlour feeding, AID = Automatic ID system, ATS = Automatic teat sprayers, EMM = Electronic milk meters, NEMM = Non-electronic milk meters, MDL = Milk diversion or Dump line, AMD = Automatic mastitis detection, ADS = Automatic drafting system, BGC = Backing gates in the collecting yard, VSVP = Variable speed vacuum pump, VSMP = Variable speed milk pump, EGP = Entrance and exit gates controlled from pit

^1^ Two herds removed due to unknown parlour type, 8 herds removed due to unanswered question
